# Microbiological and Physicochemical Assessment of Untreated Borehole Water in Residential Areas of Kubang Kerian, Kelantan

**DOI:** 10.21315/mjms-07-2025-570

**Published:** 2025-12-31

**Authors:** Wan Abdul Wahab Wan Nor Amilah, Isatou O Jah, Gillian Steffy George Lojuyo, Noor Jamil Noor Izani

**Affiliations:** School of Health Sciences, Universiti Sains Malaysia, Health Campus, Kubang Kerian, Kelantan, Malaysia

**Keywords:** borehole water, faecal coliforms, Escherichia coli, groundwater quality, Kelantan, public health

## Abstract

**Background:**

Due to concerns regarding the quality of municipal water in Kubang Kerian, Kelantan, Malaysia, many residents rely on borehole water, which is often untreated and potentially unsafe. In this study, we assess the microbiological and physicochemical quality of borehole water from household outdoor sources and examine potential risk factors in this regard, including borehole depth and the proximity to sewage systems.

**Methods:**

Thirty untreated borehole water samples were collected from residential households and analysed using standard microbiological and physicochemical techniques. Heterotrophic plate count (HPC), total coliforms, faecal coliforms and *Escherichia coli* were determined. Physicochemical parameters included pH, turbidity and free residual chlorine. Associations between microbial quality and borehole characteristics were assessed using Fisher’s exact test.

**Results:**

While 70% of samples met the Malaysian Raw Water Quality Criteria (RWQC), 30% showed microbiological contamination, including three with *E. coli*. All samples had acceptable pH and turbidity levels, though residual chlorine was absent. None of the boreholes complied with the recommended minimum distance from sewage systems, and only four met the minimum depth standard. No significant associations were found between contamination and borehole depth or proximity to sewers (*P* > 0.05).

**Conclusion:**

Despite meeting physicochemical standards, a notable proportion of the borehole samples were microbiologically unsafe. These findings underscore the need for improved borehole construction practices, routine monitoring and public education to reduce waterborne health risks.

## Introduction

Access to safe and clean water is essential in maintaining public health. However, in many developing regions, including parts of Malaysia, access to treated and reliable water sources remains a persistent challenge. Kubang Kerian, a rapidly expanding township located approximately 7 km from Kota Bharu, Kelantan, frequently experiences issues with municipal water quality. Residents have reported cloudy and rusty municipal tap water due to extensive land development in the surrounding areas of the Kelantan river, leading many households to turn to borehole water as an alternative source (1).

Contaminated water sources pose significant health risks, particularly to vulnerable populations such as children, the elderly and the immunocompromised. Exposure to unsafe drinking water has been linked to gastrointestinal infections, neurological complications and reproductive health issues (2, 3). Globally, more than 2 billion people are affected by water scarcity, and approximately 1.8 billion consume water contaminated with faecal matter, resulting in hundreds of thousands of preventable deaths each year due to diseases such as cholera, dysentery, and typhoid (4, 5).

The quality of borehole water is influenced by several factors, including construction methods, maintenance and environmental conditions. Boreholes constructed without regulatory oversight and those located too close to sewage systems or waste disposal sites are especially prone to microbial contamination (6, 7). In Kelantan, despite the region’s high annual rainfall, access to safe drinking water remains problematic. The state records the highest incidence of typhoid fever in Malaysia (8) and has experienced multiple outbreaks of cholera (9).

Previous studies in Kelantan have primarily focused on surface water contamination. However, these findings cannot be directly extrapolated to groundwater sources, such as boreholes, due to differences in contamination pathways and hydrogeological conditions. Boreholes, while often perceived as cleaner than the municipal water source due to their clarity, can still be contaminated by faecal infiltration, particularly when constructed at shallow depths or near septic systems (10, 11). Thus, relying on surface water microbial data may underestimate the true risk posed by borehole sources.

Given the widespread use of borehole water in Kubang Kerian, there is a need to assess its safety systematically. In this study, we aim to evaluate the microbiological and physicochemical quality of the untreated borehole water used by households and examine potential risk factors, such as borehole depth and proximity to sewage systems. The findings are expected to support public health efforts by informing regulatory policies, promoting safer construction practices and guiding community education on water safety.

## Methods

### Study Design and Sampling

This cross-sectional pilot study was conducted between April and July 2024 in various residential zones of Kubang Kerian, Kelantan (6°05′N, 102°17′E). A total of 30 untreated borehole water samples were collected from outdoor household outlets using a convenience sampling method. Each sample was obtained directly from the main outlet pipe connected to the borehole system before any point-of-use treatment.

Water samples were collected in sterile 1,000-mL Duran bottles, stored in insulated cooler boxes and transported to a laboratory within 2 to 5 h for analysis. A pre-sampling checklist was used to collect household-specific data, including borehole depth, distance from sewage systems and general management practices. Although formal ethical approval was not required, verbal consent was obtained from residents before sample collection.

### Microbiological Analysis

Microbiological quality was assessed using the membrane filtration technique. For each sample, 100 mL of water were filtered through a sterile 0.45-μm cellulose acetate membrane in triplicate. The membranes were placed on agar media plates for colony enumeration. Nutrient agar was used to determine heterotrophic plate count (HPC), and MacConkey agar was used to determine total and faecal coliforms. Plates were incubated at 37°C for total coliforms and HPC and at 44.5°C for faecal coliforms. Colonies were counted manually using a hand lens. Plates exhibiting excessive growth were marked as “too numerous to count” (TNTC). Faecal coliforms were further confirmed by subculturing pinkish colonies from MacConkey agar on eosin methylene blue (EMB) agar after 48 h. Colonies displaying a characteristic green metallic sheen were presumptively identified as *E. coli* and then sub-cultured on nutrient agar for purification and confirmed via standard biochemical tests (12).

### Physicochemical Analysis

Three key physicochemical parameters were analysed: pH was measured using a calibrated Hanna instrument 2211 pH/oxidation-reduction potential (HI 2211 pH/ORP) meter; turbidity was assessed with a calibrated turbidimeter, with measurements being performed in triplicate, and free residual chlorine was determined using the N-diethyl-p-phenylenediamine (DPD) colorimetric method with an Hach digital reactor 890 colorimeter. The presence of residual chlorine was indicated by a colour change.

### Data Analysis

The microbiological and physicochemical results were analysed based on the 2010 Malaysian National Water Quality Standards, Raw Water Quality Criteria (RWQC) (13) and World Health Organisation (WHO) guidelines (14) (Table 1). Statistical analysis was conducted using the Statistical Package for the Social Sciences (SPSS) software. Fisher’s exact test was used to assess the association between microbial contamination and borehole characteristics, specifically depth and proximity to sewage. A *P*-value of < 0.05 was considered statistically significant.

## Results

**Figure 1:**
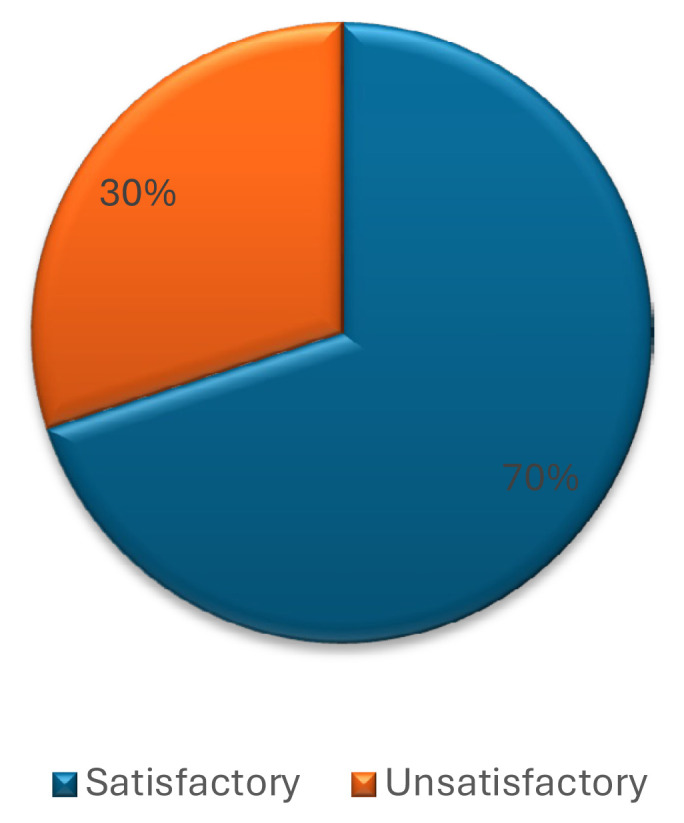
The percentage of the microbiological quality of the borehole water samples based on the Malaysian RWQC 2010

Figure 1 illustrates the proportions and distributions of samples with satisfactory and unsatisfactory microbiological quality. Of the 30 borehole water samples collected, 70% (21/30) met the Malaysian RWQC for satisfactory microbiological quality and were considered safe for drinking. As shown in Table 2, 80% of samples had HPC counts below 1,000 CFU/mL, 93.33% were within the limits for total coliforms (< 5,000 CFU/100 mL), and 96.7% were within the limits for faecal coliforms (< 5,000 CFU/100 mL). A small number exceeded these thresholds, indicating potential health risks. *E. coli* was detected in three samples. According to WHO and Malaysian standards, *E. coli* should be 0 CFU/100 mL, or absent from water intended for drinking, including untreated sources (Table 1).

The physicochemical analysis results showed that 28 samples had turbidity levels below 5 nephelometric turbidity units (NTU) and that only two exceeded this limit. Residual chlorine was undetectable or below 0.2 mg/L in all samples, which is consistent with untreated groundwater. All samples had pH values between 5.8 and 7.8, which were within the acceptable RWQC range. Overall, the physicochemical parameters met Malaysian standards.

Household checklist data revealed that none of the boreholes met the WHO-recommended minimum distance of 30 m from sewage systems. Regarding borehole depth, only four out of the 30 boreholes reached the recommended depth of more than 30 m, while 18 were shallower than recommended and depth information was unavailable for the remaining eight households (Table 3). Statistical analysis using Fisher’s exact test (Table 4) examined the association between microbiological water quality and household-specific factors (borehole depth and distance from sewage). Among the 22 households with complete data, no significant associations were found between microbiological contamination and either borehole distance from sewer systems (*P* = 0.697) or depth (*P* = 1.000), as both *P*-values exceeded the 0.05 threshold.

## Discussion

Borehole water is perceived as clearer compared to the municipal water sources, although its quality remains largely unknown. This raises concerns about contamination, particularly with faecal indicators such as *E. coli*. Thus, this study was intended to assess the microbiological and physicochemical quality of borehole water in Kubang Kerian, Kelantan, and identify the factors influencing water safety. As the water was sampled directly from the borehole outlets before any point-of-use treatment, the results reflect the microbiological and physicochemical quality of raw groundwater available to households.

We evaluated HPC, total and faecal coliforms and physicochemical parameters (pH, turbidity and free residual chlorine) in 30 borehole samples. The results showed that most of the household outdoor borehole water sources complied with the Malaysian RWQC and were deemed safe for use. Nine samples exceeded the acceptable limits for HPC, total coliforms and faecal coliforms, with *E. coli* being detected in three, indicating the three samples were severely polluted and microbiologically unsafe as sources of drinking water (15). Coliforms and heterotrophic bacteria indicate poor hygiene, while *E. coli* and faecal coliforms suggest faecal contamination (16).

In Kelantan, 49% of groundwater samples contained total coliforms, and 14% contained *E. coli* (9). Ideally, faecal indicator bacteria and total coliforms should be absent from raw drinking water sources, as their presence may indicate faecal contamination and potential health risks, even after treatment. These results were consistent with findings from Portugal (18.8%) (4) and Ethiopia (20.3%) (17), whereas in Ghana, 85% of borehole samples were contaminated (7). The presence of *E. coli* highlights the risk of gastrointestinal illness, especially from pathogenic strains such as *E. coli* O157 and O104 (7, 18).

Physicochemical parameters influence microbial survival. The pH level is critical for various biological processes and should be within a suitable range, indicating that water is safe and acceptable for drinking. Turbidity measures water clarity and the presence of suspended solids and colloidal matter in water. The latter may be due to dredging activities or the proliferation of microorganisms, and elevated turbidity levels can result in ineffective water treatment. Free residual chlorine is used to disinfect water and prevent contamination, though borehole water, which is typically sourced from underground, is expected to be free of chlorine (19).

In our study, most samples complied with RWQC, with turbidity and residual chlorine levels below the expected limit. Only two showed high turbidity (≈ 10 NTU), potentially due to fine sediments. These findings align with studies conducted in Kangar (Malaysia) (20), Mahikeng (South Africa) (7) and Nigeria (21), but differ from the unsatisfactory results reported in Dhaka, Bangladesh (22). As the water was sampled directly from the borehole outlets before any point-of-use treatment, the results reflect the microbiological and physicochemical quality of raw groundwater available to households (7, 20).

Although previous studies have reported that shallow borehole depth and proximity to sewage systems are associated with increased microbial contamination risk (10, 11), our analysis did not reveal statistically significant associations between these factors and microbial water quality. This finding stands in contrast with research conducted in rural villages of Mohale Basin, in Lesotho, which identified significant correlations (23). The lack of significance may be attributed to limited sample size and low variability in borehole compliance, as most did not meet the recommended depth or distance standards. Additionally, unmeasured confounding factors, such as construction quality, surrounding land use and geological differences, may have influenced contamination risk more strongly than depth or distance alone. Poor construction and failure to follow guidelines may contribute to contamination (7). Future studies with larger samples and more detailed borehole profiling are needed to confirm these trends.

Additional risk factors included the presence of livestock near boreholes, the use of fertilisers near shallow wells and rusted household pipes connected via electric pumps (18). Although most samples met the RWQC, untreated borehole water should not be consumed directly. Microbiological and physicochemical quality varied between households, likely due to geological differences. Samples that failed to meet the RWQC are unsuitable for use, even after treatment. Thus, households using borehole water must adopt strict safety practices to avoid bacterial infections. All borehole water should be treated before use. Effective household methods include chlorination, boiling and filtration (carbon, sand or ceramic).

To ensure safety, residents should follow regulatory standards when constructing boreholes, including appropriate site selection and distance from sources of contamination. Regular inspection and maintenance are crucial to prevent leaks in the boreholes and microbial intrusion. Public education on safe water practices, as well as water, sanitation and hygiene principles is vital (24). This study’s findings underscore the urgent need for the stricter enforcement of regulations regarding borehole construction, especially in residential areas near sewage systems.

Increasing community awareness and ongoing research will help improve water quality and reduce waterborne disease risks in the region (25). Study limitations include the small sample size (30 samples), limited geographic coverage and focus on the selected microbial and physicochemical parameters. Expanding the scope to include more households and a broader range of contaminants will provide a more comprehensive assessment. In this study, we tested only untreated samples, and further research should include treated sources, which should be compared against WHO and national standards. In addition, future research should incorporate the assessment of potentially toxic elements, such as heavy metals, along with microbial and physicochemical monitoring, to provide a more comprehensive health risk evaluation of borehole water in Kelantan.

## Conclusion

In conclusion, while most borehole water samples in Kubang Kerian met physicochemical standards, a significant portion showed microbiological contamination, posing potential health risks. These findings underscore the importance of ensuring safe borehole water through strengthened regulations; community education; routine monitoring and further research that includes seasonal variation, treated versus untreated sources and broader geographic coverage. It is also crucial to develop targeted interventions to sustain a clean and safe water supply for community consumption.

## Figures and Tables

**Table 1:** The reference values for microbiological and physicochemical comparison by the WHO and Malaysian National Water Quality Standards (13, 14)

Parameter (unit)	WHO standard	Malaysian standard	

	Drinking water quality	Raw water quality	Drinking water quality
Heterotrophic plate count (CFU/mL)	100	100 to 1000	500
Total coliform (CFU/100 mL)	0	5000	0
Faecal coliform (CFU/100 mL)	0	5000	0
*E. coli* (CFU/100 mL)	0	-	-
pH	6.5 to 8.0	5.5 to 9.0	6.5 to 9.0
Turbidity (NTU)	5	1000	5
Free residual chlorine (mg/L)	0.2 to 5.0	-	0.2 to 5.0

CFU = colony-forming unit; NTU = nephelometric turbidity unit

**Table 2:** Microbial water quality assessment as per the Malaysian RWQC

Microbial count	No. of samples	Percentage (%)	Category
**HPC (CFU/mL)**			
< 1,000	24	80	Satisfactory
≥ 1,000	6	20	Unsatisfactory

**Total coliform (CFU/100 mL)**			
< 5,000	28	93.33	Satisfactory
≥ 5,000	2	6.67	Unsatisfactory

**Faecal coliform (CFU/100 mL)**			
< 5,000	29	96.7	Satisfactory
≥ 5,000	1	3.33	Unsatisfactory

**Table 3:** Distance of the borehole from the sewer and its depth

	No. of samples (*n* = 30)	Satisfactory (*n* = 20)	Unsatisfactory (*n* = 10)
**Distance from sewer**			
< 5 ft (< 2 m)	0	0	0
5 to 10 ft (2 to 3 m)	2	1	1
10 to 20 ft (3 to 6 m)	8	6	2
> 20 ft (> 6 m)	18	12	6
Unknown	2	1	1

**Depth of borehole**			
<10 m	5	2	3
10 to 20 m	9	6	3
20 to 30 m	4	4	0
> 30 m	4	2	2
Unknown	8	6	2

**Table 4:** Association between microbiological quality and borehole distance from the sewer and its depth

Microbiological quality	Borehole distance from the sewer, *n* (%)				*P-value

	5 to 10 ft	10 to 20 ft	> 20 ft		
Satisfactory, *n* = 15	1 (6.6)	4 (26.7)	10 (66.7)		0.697
Unsatisfactory, *n* = 7	1 (14.3)	2 (28.6)	4 (57.1)		

	**Borehole depth, ** ***n*** ** (%)**				* ***P*** **-value**

	**< 10 m**	**10 to 20 m**	**20 to 30 m**	**> 30 m**	

Satisfactory	2 (13.3)	6 (40)	3 (20)	4 (26.7)	1.000
Unsatisfactory	2 (28.6)	3 (42.9)	0 (0)	2 (28.6)	

* Tested using Fisher’s exact test
